# The Integrative Effects of Leading by Example and Follower Traits in Public Goods Game: A Multilevel Study

**DOI:** 10.3389/fpsyg.2018.01687

**Published:** 2018-09-07

**Authors:** Huiqing Qiu, Youlan Zhang, Gonglin Hou, Zhongming Wang

**Affiliations:** ^1^School of Management, Zhejiang University, Hangzhou, China; ^2^Institute of Cognitive Neuroscience and Department of Psychology, Zhejiang Sci-Tech University, Hangzhou, China

**Keywords:** leading by example, personal traits, multilevel study, public goods game, leadership

## Abstract

As an important way to understand leadership based on voluntary contribution mechanisms, the importance of leading by example to teamwork is becoming more and more evident in recent years. However, existing theories based on signaling and reciprocity perspectives, respectively, provide incomplete theoretical explaining. This study adds clarity by conducting a cross-level study that indicates a possible integrative framework of both signaling and reciprocity perspective on leading by example. Results were using data gathered from 130 Chinese college students, which were allocated into one baseline group and three experimental groups. A hierarchical model was used to examine the effects of leading by example on different levels. It is found that leading by example has positive effects on the cooperation of followers on both the group level and the individual level. Risk attitudes have positive effects on the cooperation of followers while trust attitudes have negative effects. Our findings suggest that both leading by example and personal traits significantly influence cooperation but on different levels. It also reminds us that a more systematic way to understand leadership is needed.

## Introduction

Teamwork is becoming more and more crucial in our daily work including temporary cooperation and some large-scale projects. Based on the standard economic theory, individuals will always be obsessed with their personal interests and tempted to free-ride on the contributions of others. Scholars also argue that it is difficult and costly to sustain high quality cooperation (Chen et al., 2015; Pereira and Lenaerts, 2015). However, it is suggested that leading by example promotes group members’ cooperation in the context of a lack of institution and authority, where they are fully incentivized to be free riders (Fehr and Gächter, 2000; Romano and Yildirim, 2001; Levati et al., 2007). Therefore, scholars propose that leaders have to rely on voluntary leadership, usually in the form of leading by example to promote the well-functioning of teams in the absence of contractual relationships and hierarchical authority (Levati et al., 2007; Yaffe and Kark, 2011). The inconsistency between standard economic theory prediction and observed phenomena indicates that both empirical and theoretical work are needed to clarify the underlying process of voluntary leadership. In addition, understanding the processes and underlying mechanisms that link leading by example and the cooperation of followers is vital, because they also allow leaders to create specific interventions to best leverage leadership for positive effects on group members and performance. However, extant research still does not clearly reveal whether leading by example exerts positive or negative influence on team members. Therefore, we conducted this study to explore the mechanism of leading by example.

Although scholars agree on the importance and effectiveness of leading by example, there is still a lack of consensus on an integrated theoretical framework for comprehending the effects on group members’ behaviors and group performance (Fehr and Gächter, 2000; Moxnes and van der Heijden, 2003; Güth et al., 2007; Levati et al., 2007). Economic and psychological theory provide two different perspectives based on observed data, which are “signaling perspective” and “reciprocity perspective.” In the case of symmetric-information as well as full information settings, group members are all well informed about the marginal return of their cooperation. But in the case of asymmetric-information settings, some group members may be better informed about the marginal return of their cooperation. Thus, researchers who believe that signaling perspective is of primary importance argue that leading by example in asymmetric-information is superior to symmetric-information by committing effort first to signal information about the marginal return of cooperation (Hermalin, 1998). Therefore, much attention is paid to the information transferring, especially in the presence of asymmetric information (Chen and Komorita, 1994). As a group level phenomenon, it mainly focuses on the process of how leaders credibly communicate their information to other group members. On the other side, the reciprocity perspective suggests that the cooperation of later movers is significantly and strongly correlated with that of earlier movers (Güth et al., 2007; Levati et al., 2007; Potters et al., 2007; Gächter et al., 2012; Arbak and Villeval, 2013). The signaling perspective is limited in explaining how leading by example works when there is no asymmetric-information, while the reciprocity perspective does not exclude the effect of signaling.

Scholars arguing in favor of signaling perspective claim that in an asymmetric-information environment the signaling function is the key to understanding leading by example (Hermalin, 1998; Potters et al., 2005). On the other hand, non-pecuniary factors such as reciprocity may cause leading by example to be effective in a full-information context (Fehr and Gächter, 2000; Romano and Yildirim, 2001). In summary, these two theoretical perspectives afford an incomplete theoretical interpretation for both the group and individual levels, leaving a theoretical gap to fill. Thus, a new theoretical interpretation of observed data integrating both the group level and the individual level is necessary.

In the public goods experiment, leading by example is defined as the leaders determine their cooperation to the public goods before other group members reveal theirs. All subjects as followers were unaware the identity of the group leaders, who were played by experimenters. By sampling leader contribution behavior from two predetermined sets, we controlled the cooperative level of leading by example. All the experimentation and data gathering processes were conducted on the platform of z-Tree, which is authored by Fischbacher (2007). We explored the effects of individual cognition on other subjects’ cooperation by taking their Machiavellianism, trust and risk attitudes into account. These personal traits are believed to be related to how individuals process the information received during the experiments, which is helpful in adding clarity to the cognitive process on the individual level.

Finally, this research extends previous studies at least in four ways. First, through a hierarchical linear model (hereafter, HLM), we examine the effects of both leading by example and personal traits on group members’ cooperative behavior. Therefore, considering the incomplete theoretical explanation provided by the signaling perspective and reciprocity perspective, this study furthers the scope of leading by example by providing a comprehensive framework on both the group level and the individual level. Second, we explore how good leaders and poor leaders affect group cooperation by manipulating the level of leading by example. We specifically show that bad leadership is even worse than no leadership, implying that researchers should also pay more attention to the negative effects of bad leadership. Third, this research stresses the importance of the cognitive process by focusing on personal traits, which affect how people evaluate the behavior of others. Previously, researchers noticed that followers in a public goods game act as conditional cooperators (Chaudhuri and Paichayontvijit, 2006; Chaudhuri, 2011), suggesting that individuals perceive and interpret received information differently. That reminds scholars that cognition plays an important role when leadership influences followers. Finally, some confounding factors were excluded from our study to create better settings for observing the effect of leading by example. Although never seriously considered previously, we propose that leaders are likely to react to the cooperation of followers. Thus, we controlled leading by example behaviors from a fixed distribution to exclude the underlying confusion caused by the dynamic interaction between leaders and followers.

### Literature Review and Hypotheses

#### Leading by Example and Cooperation

Leading by example is a continually growing field. Hermalin (1998) proposed leading by example as an economic theory of leadership, which is defined as leaders contributing before followers. It has been proven as an effective way to promote cooperation and improve group performance (Hermalin, 1998; Vesterlund, 2003; Potters et al., 2007). Among different perspectives of leadership, it provides us with an empirical way to explore the mechanism of how leaders influence group members. As a normative theory, it provides a mathematical prediction that leadership will prompt followers not to be free riders and coordinate onto an equilibrium significantly away from the Nash Equilibrium, which invites further study. However, few field studies have been conducted because of the complexity and difficulties of gathering data. Scholars prefer using experimentation to observe how leading by example impacts cooperation, especially with the voluntary contribution mechanism (Gonzalez et al., 2005; Gächter et al., 2012).

The theoretical debate between signaling perspective and reciprocity perspective has been going on for years. The literature of previous experiments in this field provides robust evidence that leading by example has positive effects on the cooperation of followers compared to a situation without leadership (e.g., Potters et al., 2007; Gächter et al., 2012; Dannenberg, 2015), supporting the ideal of signaling as the fundamental mechanism (Hermalin, 1998). Scholars have also presented evidence confirming that leading by example is caused by reciprocity (Meidinger and Villeval, 2002; Moxnes and van der Heijden, 2003). Using the sequential prisoners’ dilemma game, Clark and Sefton (2001) reveal that the first mover’s choice of cooperation is the most important variable influencing the second mover’s cooperation, supporting the idea that reciprocation has positive effects on cooperation. It also begs the question whether leading by example is somehow confounded with reciprocation in social dilemma, because there is no direct evidence denying that signaling is valid. In fact, even researchers adhering to the idea of reciprocity also admit that the effect of leading by example is significant (Meidinger and Villeval, 2002; Moxnes and van der Heijden, 2003). Given the mixed evidence on the effectiveness of leading by example, the question arises how the mechanisms under the process of leading by example encourage followers to be cooperative.

Based on existing theories and empirical evidence, scholars have tried to address many important research questions surrounding leading by example via exploring predictors and outcomes from different levels. To examine which theoretical perspective provides a better understanding of leading by example, Potters et al. (2007) conducted an experimental study to compare the effects of leading by example in environmental settings both with and without asymmetric-information. They found that leading by example has no impact on the cooperation of followers when all information about returns are commonly known. Empirical evidence also suggests that the cooperative behavior of later movers is significantly and strongly correlated with those of the earlier movers (Güth et al., 2007; Levati et al., 2007; Potters et al., 2007; Gächter et al., 2012; Arbak and Villeval, 2013). Similar findings have been reported in the sequential prisoners’ dilemma game, showing that the second mover tends to be cooperative when the first mover also chooses to be cooperative (Clark and Sefton, 2001). That supports the idea that leading by example influences group members via signaling. Meanwhile, after observing for evidence of reciprocity that is consistent with the existing literature, the possibility that followers are incentivized by social motives cannot be ruled out.

Overall, previous literature implies that leading by example effectively improves the cooperation of followers, leaving the underlying mechanism to be explored. The existing literature is silent about the possibility that both the group and individual levels’ factors function together to demonstrate leading by example. Signaling and reciprocity both offer an explanation for the mechanism of leading by example on followers on different levels. The signaling perspective establishes theory on the group level by focusing on information structure and group dynamics. The reciprocity perspective provides an individual level interpretation using personal traits and social preferences. The continuing tension between these two theoretical perspectives is due to the lack of cross-level empirical research. Therefore, this study examines the influence of leading by example on both group and individual level dependent variables. Therefore, we postulate in a new combined fashion that leading by example facilitates the cooperation of followers both on the group level and the individual level.

***Hypothesis 1:*** Leading by example will positively influence the contributions of the followers.
***Hypothesis 2:*** Leading by example will positively influence the payoff of the a) teams, b) leaders and c) followers.

#### Personal Traits

Existing research makes it clear that individuals often react differently to the same leader. It is suggested that many people in public goods experiments act as “conditional cooperators,” whose cooperation is positively correlated with their belief in the cooperation of others (Chaudhuri and Paichayontvijit, 2006; Chaudhuri, 2011). This conclusion clarifies how followers, as active information processers, perceive the leadership information that will influence their attitudes and behaviors in supporting the team in achieving its social and economic goals. Personal traits are related to how followers perceive and comprehend leadership, which has been proposed as a vital factor influencing the effects of leading by example (Gächter et al., 2004; Gächter et al., 2012; Drouvelis and Nosenzo, 2013). For example, empirical studies show that economic education is negatively correlated with cooperation (Marwell and Ames, 1981; Frank et al., 1993). Existing literature primarily examines the relationship between personal traits and leader willingness to contribute, which neglects the importance of the role of followers. Therefore, traits possessed by followers might influence the cooperation of followers.

In accordance with previous studies, three measured variables have been selected to explore the link between personal traits and the effects of leading by example on followers. The three variables which have been commonly studied in evaluating the inclinations of followers are Machiavellianism, trust attitudes and risk attitudes.

Machiavellianism is related to cooperativeness, which has been systematically studied by Christie and Geis (1970), who developed the Machiavellianism scale (Mach-IV) as a useful instrument to measure an individual’s tendency toward Machiavellian behavior. Many studies focus on the predictive power of the Machiavellianism scale by comparing how the behavior of subjects with both high and low Machiavellianism scale scores differs in experiments. Furthermore, the predictive validity of such studies has been acknowledged through further research (Fehr et al., 1992). Individuals who have a high level of Machiavellianism are less likely to trust their partners and are more likely to be opportunists. For example, one experimental study shows that Machiavellianism predicts an individual’s propensity to reciprocate less or to defect more in an anonymous bargaining game (Gunnthorsdottir et al., 2002). It is likewise shown that subjects with low Machiavellianism scores tend to make more contributions than those who score high (Gächter et al., 2012). Therefore, this study proposes the following hypothesis to confirm a negative relationship between Machiavellianism and cooperative behavior.

***Hypothesis 3a:*** Machiavellianism will negatively influence the cooperation of followers.

Scholars are interested in trust attitudes because the voluntary cooperation of economic agents simply can’t be formally enforced and they argue that trust is a determinant of cooperation. For instance, it is found that trust attitudes are significantly and positively correlated with cooperative behavior in a one-shot public goods experiment (Gächter et al., 2004). Drouvelis and Nosenzo (2013) provide support for the theory that trust attitudes are positively related to successful cooperation. Thus, we postulate that high level trust attitudes promote the cooperation of followers with the following hypothesis.

***Hypothesis 3b:*** Trust attitudes will positively influence the cooperation of followers.

Risk attitude refers to an individual’s willingness to take risks in general and remains stable across all contexts. Experiments have shown risk attitude to be a good predictor of potentially risky behaviors such as managing stocks, occupational choice and lottery participation (Dohmen et al., 2011). The growing literature sheds light on the importance of risky behavior because the heterogeneity of risk attitudes systematically leads to differences in economic decisions among different individuals. Although previous research suggests that self-reported risk attitudes are not predictive of neither leaders’ (Gächter et al., 2012; Drouvelis and Nosenzo, 2013) nor followers’ cooperation (Gächter et al., 2012), this study nonetheless expects a positive correlation. It is likely that previous studies confused the effect of risk attitudes with the effects of learning by calculating the average contributions of the followers through many rounds. Via Hierarchical Linear Modeling (HLM), we explore the relationship between risk attitudes and every contribution decision. Since a positive response to leading by example is a risky decision related to the ultimate payoff, it is expected that individuals who are more willing to take risks will be more cooperative in public goods game (hereafter, PGG) experiments. As a result, the following hypothesis regarding risk attitudes has been proposed.

***Hypothesis 3c:*** Risk attitudes will positively influence follower cooperation.

## Materials and Methods

### Public Goods Game (PGG) Paradigm

As a widely adopted behavior economic research paradigm, PGG based on the voluntary contribution mechanism assists in observing leading by example by controlling experiment conditions (Güth et al., 2007; Levati et al., 2007). It provides precise definitions of leading by example, the cooperation of followers, incentive mechanisms and information structure. According to Isaac et al. (1985), the game involves a partnership of *n* (2 ≤*n*) subjects who are supposed to share equally in the payoff depending on their individual decisions and a designed productivity parameter β for *t* (t = 1,…, T) rounds. In every round of the game, each subject is endowed with *e*_i,t_ tokens, which can be either reserved or used to contribute to the group activity. Subject *i* has to contribute *c*_i,t_ tokens to the group activity, which satisfies 0 ≤ c_i,t_ ≤ e. Denote u_i,t_ as the ultimate earnings of subject *i*’s earning in round *t*, and calculate it by using the following equation:

(1) $$\begin{array}{c} u_{i,t}=e_{i,t}-c_{i,t}+\beta c_{t}, \end{array}$$

where c_t_ = $\sum_{i=1}^{n}$c_i,t_ and 1/n < β < 1. Following experimental design in previous literature (Güth et al., 2007; Dannenberg, 2015), β is set as 0.4 in this study. Thus, cooperation is defined as donated tokens as well as the contributions of subjects. The dominant strategy of each rational player is to contribute absolutely nothing to the group activity in order to get *e* as the ultimate earning in each round. However, only if the subject donates all the tokens received in each round, will the socially efficient outcome ($\sum_{i=1}^{n}$u_i,t_ = nβe) be reached. Those who contribute more than average are defined as “cooperators”; those who contribute less than average are defined as “free riders.” By reducing their contributions to the group activity, free riders benefit themselves and wreak havoc in the groups. Previous empirical studies show that monetary (Fehr and Gächter, 2000; Masclet et al., 2003), information about the presence of conditional cooperators (Chaudhuri and Paichayontvijit, 2006); expressions of disapproval and preference (Masclet et al., 2003); assortative matching subjects (Gächter and Thöni, 2005; Gunnthorsdottir et al., 2007); and leading by example (Pogrebna et al., 2011; Dannenberg, 2015) are all highly related to sustaining high contributions to public goods. To sum up, the PGG paradigm provides clarity in exploring the mechanism of group members’ interaction, which is, as yet, not fully understand by scholars, as is evident from the existing literature.

Following the experiment design without punishment in previous literature (e.g., Gächter et al., 2012; Drouvelis and Nosenzo, 2013; Dannenberg, 2015), repeated PGG paradigm has been accepted as a valid way to study leading by example. This is because that adapting one-shot PGG with leadership may introduce the end-of-game effect (Keser and van Winden, 2000; Gonzalez et al., 2005), which strongly drives followers to be free-riders because they believe that they won’t get punished or prized from their leaders any more. Moreover, it takes time for leaders to establish their reputation as a signal both in experiments and realistic, which is better to use repeated PGG with leading by example for simulation. A slightly different form of the PGG paradigm with leading by example as adopted by this empirical study includes two stages. First, *leader, l*, reveals his/her contribution *C*_l,t_ to all followers. Second, *followers, f*, simultaneously decide on their own contributions *C*_f,t_. Applying game theory and backward induction, rational followers will contribute nothing to reach their maximal ultimate earnings, and the leader will also free-ride because he/she can anticipate this strategy. If both leader and followers contribute nothing to the group, the Nash equilibrium has been reached. Therefore, all subjects are actually motived to be free riders due to rationality. Thus, the PGG paradigm offers a theoretical baseline drawn from game theory, which can be compared to the followers’ actual behaviors.

According to existing literature, it is important to identify and comprehend individual variations of cooperative behavior in the PGG (Fehr and Gächter, 2000; Romano and Yildirim, 2001; Levati et al., 2007). Since different followers behave differently for different leaders, scholars conclude that personal traits are helpful for predicting people’s behaviors (Fehr et al., 1992; Frank et al., 1993; Dohmen et al., 2011) even in the PGG paradigm with or without leading by example (Gächter et al., 2012; Drouvelis and Nosenzo, 2013; Corr et al., 2015). Thus, this study takes three important personal traits: Machiavellianism, trust attitudes and risk attitudes, into account in order to understand how individuals actively react to leading by example.

### Ethics Approval Statement

The study is approved by ethic committee of human study in Zhejiang University Global Entrepreneurship Research Centre and Zhejiang Sci-Tech University. The data was volunteered by our studies participants. All the participants provided written informed consent after they were provided ample information about the “purpose, procedure, risks and discomforts, benefits and confidentiality” of this study. We also assured them that their response were private and anonymous. The procedure followed the guidelines of human research from ethic committee in Zhejiang University and Zhejiang Sci-Tech University.

### Pilot Study

Previous literature suggests that, as the PGG experiment progress, the cooperation of followers which leading by example initiated will not remain at a high level. Because leaders always make decisions earlier than followers, it follows that the personal gain of leaders will be lower than that of followers (e.g., Levati et al., 2007; Gächter et al., 2012; Figuieres et al., 2012). Leaders will consider that others get a free ride and thus gradually decrease their contributions (Gächter and Renner, 2003). In addition, Gächter et al. (2012) distinguish different levels of leader cooperation. Therefore, in order to distinguish the different levels of cooperation, and ensure the validity of the experiment software (z-Tree), we undertook a pilot study before conducting formal experiments.

We invited 40 university students, 24 men and 16 women, to participate. None of them were economic majors. Each participant was assigned randomly to a four-member temporary group, and one of the four was appointed as the leader. Following Güth et al. (2007) and Gächter et al. (2012), each four-member group took part in a 30 turn sequential voluntary contribution game experiment. At the beginning of each turn, each member had 20 tokens. The leader then donated some of his own tokens to the public pool (the amount was decided solely by the leader). All the followers were informed of the contributions of the group leader; then they also made a decision whether or not and how much to donate. There were four subjects, thus *n* = 4. As stated above, β = 0.4 in this experiment. Therefore, all the tokens in the public pool were multiplied by nβ, where *n* = 4 and β = 0.4 in this experiment, then returned evenly to the four members of the group. Thus, the payoff for each turn consisted of two parts, i.e., the part returned from the public pool and the part which was not donated. At the end of every turn, all members would receive feedback which included the detailed amount of the payoff each member for that turn. All participants were informed that their payoff in the experiment would be associated with their actual pay.

**FIGURE 1:**
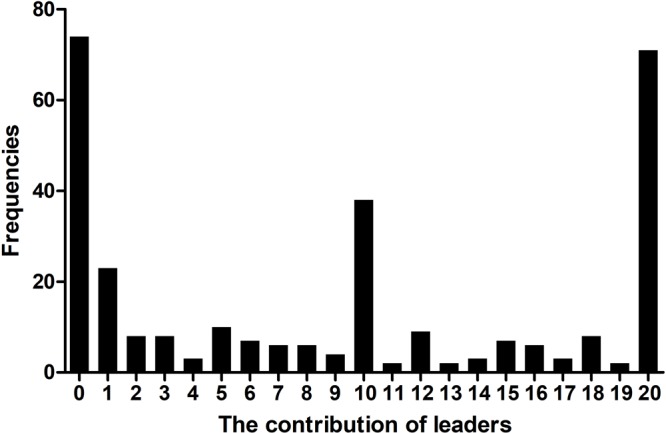
The contributions of the leaders in pilot study.

The results of the pilot study show the average contributions of the leaders was 9.08 (*SD* = 7.95), and the distribution of which was trimodal with three peak values at 0, 10, and 20 (see **Figure 1**). These results helped us operationalize different levels of leading by example-initiated cooperation: the average contributions of leaders were 0.30 on the low level, 8.27 on the medium level and 18.93 on the high level.

### Participants, Experimental Design and Procedure

One hundred and thirty university students were invited to participate in our experiment. There were 66 women and 64 men, all either current college students or graduates, none of whom were or had been economics majors. 90 of them were assigned to the condition leading by example experiment group, while 40 of them were assigned to an ordinary PGG paradigm group, which was treated as the control group. None of the participants had taken part in similar experiments. Following the experimental design in previous studies (e.g., Meidinger and Villeval, 2002; Potters et al., 2007; Gächter et al., 2012), more than 12 subjects in each session will be comparable to previous studies. Also, 30 turns and repeated measures contributed to robust consequences.

The formal experiment also adopted the same paradigm as in the pilot study, except that in this case, our assistant played the leading role in the decision-making process. In the formal experiment, the four-member group consisted of three participants acting as followers, with the assistant as the leader. Note that each of the participants finished the task by using a terminal computer located in a separate room, and none of them knew the part of the leader was assigned to our assistant.

Following Fehr and Gächter’s (2000) advice, we adopted between-group design for experiment groups to examine how leading by example influences the cooperation of followers. As we pointed out in the pilot study, the different levels of cooperation are operationalized as the average contributions of the leaders, and the low, medium, and high levels are referred as 0.30, 8.27, and 18.93, respectively. In the 30 turns process, the contributions of the leaders varied randomly, while the average number was in line with its assigned level of cooperation. For example, the contributions of the leader in group 15th (the medium level of leading by example) was 5, 10, 12, 10, 5, 5, 13, 10, 4, 2, 10, 10, 6, 10, 10, 12, 8, 10, 9, 3, 12, 6, 11, 7, 8, 10, 3, 7, 10, and 10. All other conditions were the same as in the pilot study. In the control groups, there were no leaders; each of the four members were participants, and they finished the PGG experiment together (i.e., all members donated simultaneously and received feedback at the end of each turn).

We also investigated the personality traits of each participant through questionnaires at the beginning of the experiment. We focused on three variables in this research – Machiavellianism, trust attitude and risk attitude. Considering that all the original scales were in English, we followed a back-translation procedure (Brislin et al., 1973). Two English teachers translated the original scales into Chinese, and two other English teachers translated the Chinese documents back into English in order to ensure the reliability of the translated scales.

Machiavellianism was measured by the Machiavellianism Personality Scale (MPS), a 16-item 5-point Likert scale, which was developed by Dahling et al. (2009). A sample item is “I believe that lying is necessary to maintain a competitive advantage over others” (Cronbach’s α = 0.810).

To measure trust attitude, we adapted an item from the World Values Survey (WVS), which is “Generally speaking, would you say that most people can be trusted or that you need to be very careful in dealing with people?” (Inglehart, 1997, 2004). Participants chose “most people can be trusted” or “to be very careful in dealing with people” to reveal their attitudes. We coded “You need to be very careful in dealing with people” as 0 and “Most people can be trusted” as 1.

We assessed risk attitudes by using an item from the German Socio-Economic Panel (GSOEP), which is “Are you generally a person who is fully prepared to take risks or do you try to avoid taking risks?” (SOEP Group, 2011). The participants were asked to mark from 1 to 10, where 1 standing for “very unwilling to take risks” and 10 standing for “fully prepared to take a risk.”

### Data Analysis

A manipulation check was done before doing the data analysis. We required all participants to assess the leader in a task responding to a 5-point item which was “I thought the one who donated first was selfish” (1 = totally disagree, 5 = totally agree).

Analyzing the data of this experiment was divided into two sessions. In the first session we did non-parametric tests to find out how leading by example influenced the cooperation of the groups, and how it was related to the individual payoff of the leaders and the followers.

In the second session, we applied a hierarchical linear modeling (HLM) approach to investigate the effects of leading by example and personality traits on the contributions of the followers. We used a three-level model for this research: Level 1 is the behavior level, where the variables representing the contributions of the followers; Level 2 is the individual level, which included Machiavellianism, trust attitude and risk attitude representing personal traits; Level 3 is the group level, where the contributions of the leaders (leading by example) were tested. Note that we verified the within-team agreement and null-model before beginning the HLM analysis.

## Results

### Manipulation Check

As described before, we required all participants to assess their leader in terms of selfishness. For the groups in the high, medium and low levels of leading by example, the rated scores were 4.47 (*SD* = 0.82, *N* = 30), 3.17 (*SD* = 0.87, *N* = 30), 2.33 (*SD* = 1.47, *N* = 30) respectively. ANOVA revealed that the members of these three groups have a significantly different perception of the selfishness of their leader (*F* = 28.93, *p* < 0.001).

### Non-parametric Tests

First, we calculated the average contributions of the teams and of the followers. The results indicated that the average team contribution for low, medium and high levels of leading by example conditions were 3.99, 6.37, and 8.10, respectively, while for the control group, the number was 8.14 (see **Table 1**). The Kruskal–Wallis test revealed that the difference is significant on the 0.05 level (χ^2^= 8.81, *p* < 0.03). We ran a Mann–Whitney *U* test to compare different contributions among different conditions and found that the difference between contributions varied: (a) on the high and low level of leading by example conditions, the difference was significant (*z* = 2.19, *p* < 0.05); (b) on the low level of leading by example conditions and control condition, the difference was also significant (*z* = 2.65, *p* < 0.01). These results implied that a high level of leading by example will promote cooperation (i.e., more average contributions of teams), while a low level will impair it. Thus, H1 is supported.

**Table 1:** Individual contributions regarding different conditions.

Condition	Team average		Follower average		Comparisons		
	*M*	*SD*	*M*	*SD*	Medium	Low	Controlled
High	10.81	3.26	8.10	4.34	0.76	2.19^∗^	0.27
Medium	6.77	1.30	6.37	1.73		1.78	1.44
Low	3.06	1.89	3.99	2.52			2.65^∗∗^
Controlled	8.14	2.49					

*n = 130. Contributions were computed as the average of teams and followers; Mann–Whitney U test were run to compare differences among different conditions; High, Medium, Low refer to different levels of leading by example, while Controlled refers to control conditions. ^∗^p < 0.05, ^∗∗^p < 0.01.*

On the other hand, it was noted that followers were almost impossible to free ride in the low level of leading by example condition. However, subjects still were reciprocal to other members in the group. Moreover, some followers were strongly altruistic that they would contribute regardless how few others contribute. Moreover, the margin effect of leading by example declined when the level of leading by example was already high. It indicated that it was costly to maintain cooperation, which was consistent with previous literature (Chen et al., 2014, 2015; Wang et al., 2018).

**FIGURE 2:**
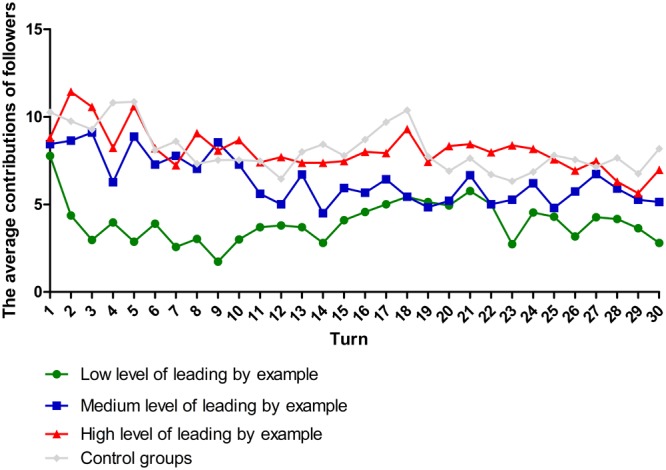
The contributions of the followers.

Second, we felt that the time frame of the PGG paradigm warranted further examination. Previous literature suggesting that end-of-game effects (Keser and van Winden, 2000; Gonzalez et al., 2005), which states that the contributions of subjects in repeated public goods game decline over time and reach their minimum when the game terminates (see **Figure 2**). Rational subjects might realize that the defection strategy will be the dominant strategy when the game is going to end, and probably choose to defect. Scholars also tried to provide a theoretical interpretation with evolutionary game theory to describe how subjects renew their strategies as the game is going on (Sasaki et al., 2015; Pereira and Lenaerts, 2017; Wang et al., 2018).

We compared the average contributions of teams under different conditions (see **Table 2**), using the Kruskal–Wallis test which indicated that the contributions under different conditions have no significant differences at turn 1st (χ^2^= 4.39, ns), but differed significantly at turn 2nd (χ^2^= 12.75, *p* < 0.01) and turn 30th (χ^2^= 10.89, *p* = 0.01).

**Table 2:** The contributions of the followers at turn 1, 2, and 30, regarding different conditions.

Condition	Turn 1		Turn 2		Turn 30	
	*M*	*SD*	*M*	*SD*	*M*	*SD*
High (H)	8.80	5.45	11.43	6.23	6.97	4.92
Medium (M)	8.43	1.94	8.63	2.67	5.13	2.62
Low (L)	7.77	3.65	4.37	3.16	2.80	2.80
Controlled (C)	10.25	2.24	9.75	3.14	8.18	4.34
Comparison	*ns*		H > M; M > L; L < C		M < C, L < C	

*n = 130. The average contributions of teams were computed at turns 1, 2, and 30; the Mann–Whitney U test was run to compare differences among different conditions; High, Medium, Low refer to different levels of leading by example, while Controlled refers to control conditions*.

In addition, the Mann–Whitney *U* tests revealed the detailed results. At turn 2nd, the difference of contributions was significant: (a) between the low and high levels of leading by example conditions, (*z* = 2.46, *p* < 0.05); (b) between the low and medium level of leading by example conditions, (*z* = 2.69, *p* < 0.01); and (c) between the low level of leading by example conditions and control conditions (*z* = 2.65, *p* < 0.01). At the turn 30th, the difference of contributions was significant: (a) between the low level of leading by example conditions and control conditions, (*z* = 2.87, *p* < 0.01); and (b) between the medium level of the leading by example conditions and control conditions, (*z* = 2.01, *p* < 0.05). These results supported conclusions above, and also indicated the “end-of-game effect.” As the turns increased and the game was going to end, followers slowly realized that the defection was a dominant strategy, and the contributions of followers decreased. However, even though the end-of-game effect might work, leading by example still exerted significant influence on the contributions of the followers after 30 turns, which provided extra evidence for supporting H1.

Third, we computed three kinds of payoffs under different conditions (see **Table 3**). The Kruskal–Wallis tests proved the significance of differences in four conditions regarding the average payoff of teams (χ^2^= 25.01, *p* < 0.01), the average payoff of followers (χ^2^= 33.02, *p* < 0.01) and the average payoff of leaders (χ^2^= 12.88, *p* < 0.01). Using the Mann–Whitney *U* tests, we compared the detailed differences among these conditions.

**Table 3:** The payoffs regarding different conditions.

Condition	Payoff of teams		Payoff of leaders		Payoff of followers	
	*M*	*SD*	*M*	*SD*	*M*	*SD*
High (H)	26.48	1.95	18.36	5.21	29.19	0.87
Medium (M)	24.06	0.78	22.56	2.08	24.56	0.35
Low (L)	21.84	1.13	24.61	3.02	20.92	0.50
Controlled (C)	24.88	1.49	24.88	1.49	24.88	1.49
Comparison	H > M; H > L; M > L; L < C		H < M; H < L; H < C; M < C		H > M; H > L; H > C;M > L; L < C	

*n = 130. Payoffs were computed as the average payoff of teams, followers and leader; the Mann–Whitney U test was run to compare differences among different conditions; High, Medium, Low refer to different levels of leading by example, while Controlled refers to control conditions*.

For the average payoff of teams, the results indicate that (a) the average payoff of teams on the high level of leading by example conditions was higher than on either the low level (*z* = 3.18, *p* < 0.01) or the medium level (*z* = 2.67, *p* < 0.01); (b) the average payoff of teams on the low level of leading by example conditions was lower than on both the medium level (*z* = 3.63, *p* < 0.01) and in control conditions (*z* = 3.45, *p* < 0.01). These results indicate that a high level of leading by example will increase the payoff of teams, whereas a low level will decrease it. Thus H2a is supported.

For the average payoff of followers, the results demonstrated that (a) the average payoff of followers on the high level of leading by example conditions was higher than on the medium level (*z* = 3.78, *p* < 0.01), on the low level (*z* = 3.78, *p* < 0.01) or in control conditions (*z* = 3.78, *p* < 0.01); (b) the average payoff of followers on the low level of leading by example conditions was lower than on the medium level (*z* = 3.78, *p* < 0.01) or in control conditions (*z* = 3.78, *p* < 0.01). These results indicate that a high level of leading by example will increase the payoff of followers, while a low level will decrease it. Thus H2b is supported.

For the average payoff of leaders, the results revealed that (a) the average payoff of leaders on the high level of leading by example conditions was lower than on the medium level (*z* = 1.97, *p* < 0.05), on the low level (*z* = 2.65, *p* < 0.01) and in control conditions (*z* = 2.80, *p* < 0.01); (b) the average payoff of leaders on the medium level of leading by example conditions was lower than in control conditions (z = 2.27, *p* < 0.01). These results indicate that a high level of leading by example will decrease the payoff of leaders, while a low level will increase it. Thus H2c is supported.

In addition, we ran an additional analysis on the differences between average payoff of followers and the average payoff of leaders. The Wilcoxon signed-rank tests demonstrated that (a) the average payoff of followers was higher than the average payoff of leaders on high (*z* = 2.80, *p* < 0.01) and medium (*z* = 2.29, *p* < 0.05) levels of leading by example conditions; (b) the average payoff of followers was lower than the average payoff of leaders on the low (*z* = 2.80, *p* < 0.01) level of leading by example conditions.

Considering the results of this analysis of the three kinds of payoffs, we conclude that leading by example will have an effect on both team and individual outcomes, thus H2 is supported.

### Hierarchical Linear Modeling Tests

To test our hypotheses that the level of the contributions of leaders and follower traits will influence the contributions of followers, we analyzed the data from the experience group using the HLM 6.0 program (Raudenbush et al., 2004). **Table 4** displays the descriptive statistics and correlations among variables used in this study.

**Table 4:** Means, standard deviations, and correlations among variables^a^.

Variable	Mean	*SD*	1	2	3	4	5
**Trait level**							
(1) Machiavellianism	2.28	0.50					
(2) Trust attitude^b^	0.67	0.47	–0.43^∗∗^				
(3) Risk attitude	6.18	2.06	–0.06	0.07			
**Behavior level**							
(4) The contribution of followers in each turn	6.15	6.61					0.27^∗∗^
(5) The contribution of leaders in each turn	9.17	7.90					

*^*a*^*n* = 90 for variable 1–3, *n* = 2700 for variable 4, *n* = 900 for variable 5. ^*b*^0 = “You need to be very careful in dealing with people, 1 = “Most people can be trusted. ^∗∗^*p* < 0.01.*

In this multilevel model, the contributions of followers in each turn was regressed on the turn effects on level 1. The estimate on level 1 revealed the turn effects on the contributions of followers. HLM estimated the between-individual effects of individual traits (Machiavellianism, trust attitude, risk attitude) on level 2 and the between-group effects of the levels of leading by example on level 3. All variables on level 1 were group-mean-centered and all variables on level 2 were grand-mean-centered (Hofmann and Gavin, 1998). Following Muthén and Asparouhov’s (2009) suggestion, we explored the between-effects of the level of the contributions of leaders and follower traits on the contributions of followers using the fixed-intercept model.

Before conducting multilevel analyses, we first examined whether the contributions of followers in each turn varied substantially within, as well as between, individuals and groups. Results of a null model showed that both a significant between-individual variance (τ*_π00_* = 17.43, χ^2^= 1553.81, *df* = 60, *p* < 0.001) and a between-group variance (*τ_β00_* = 5.25, χ^2^= 56.09, *df* = 29, *p* < 0.01) existed in the contributions of followers in each turn. The intraclass correlation coefficient (ICC) indicated that 40% of the variance in followers’ contribution amounts at was due to between-individual variability and 13% was due to between-group variability, thus justifying further cross-level analyses. **Table 5** presents the hierarchical linear modeling results.

**Table 5:** Hierarchical linear model results for the contributions of the leaders and followers’ traits predicting the contributions of the followers.

Variable	Coefficient (γ_ijk_)	*SE*	*t*
**Level 1**			
Turn γ_100_	–0.06^∗^	0.03	–2.44
**Level 2**			
Machiavellianism γ_010_	–0.25	1.01	–0.25
Trust attitude γ_020_	–2.28^∗^	0.89	2.57
Risk attitude γ_030_	0.88^∗∗^	0.20	4.45
**Level 3**			
Levels of leading by example γ_001_	1.86^∗∗^	0.58	3.23

*^*a*^This table shows results of an “intercepts-as-outcomes” analysis. Level 1, n = 2700; Level 2, n = 90; Level 3, n = 30.^∗^p < 0.05, ^∗∗^p < 0.01*.

The results from the intercepts-as-outcomes model showed that the contributions of leaders positively predicted the contributions of followers (γ = 1.86, *p* < 0.01). Thus, hypothesis 1b is supported. Moreover, the sequence of turns was negatively related to the contributions of followers, implying the end-of-game effect. These results were also consistent with conclusions we drawn in the previous section.

Since the current section focused on followers’ traits, we examined the coefficients of level 2 variables. Hypothesis 3a was not supported in that the effect of Machiavellianism was not significant (γ = -0.25, n.s.). In terms of Hypothesis 3b, we obtained a different result showing that trust attitudes negatively predicted the contributions of followers (γ = -2.28, *p* < 0.05), thus out of our expectation with hypothesis 3b. It was indicated that followers who chose “Most people can be trusted” would tend to cooperate less. The correlation between the trust attitude of followers and their average contributions was also significantly negative (*r* = -0.22, *p* < 0.05). This was an unexpected result, which raised the question of how trust interact with leading by example. We interviewed some subjects after they finished their tasks. There were two different opinions worthy of our attention. Some subjects said that they totally trusted that the leaders and other followers would be generous, which provided them opportunities for free-riding. On the other hand, some followers had a strong feeling of being betrayed when they found that some leaders or followers were free-riding. Thus, the influence of trust on leading by example should be explored further in future research. We suggested future studies to examine the underlying psychological processes associated with leading by example. Finally, the result supported hypothesis 3c that risk attitudes positively influenced followers’ contribution (γ = 0.88, *p* < 0.01).

## Discussion

Based on the effort to abstract the concept of leadership from confounding power and intuitional factors, the vital role of leading by example is well recognized in existing scholarship (Hermalin, 1998; Fehr and Gächter, 2000; Drouvelis and Nosenzo, 2013; Dannenberg, 2015). Hermalin (1998) already showed us a formal model to interpret the deviation from equilibrium predictions, which was viewed as the effectiveness of leading by example. Leaders can make a side-payment to followers, which is not directly relevant to followers’ contributions. The more leaders sacrifice, the greater followers believe the marginal return to be. Also, leaders can commit effort first to signal information about the marginal return through leading by example. Instead of free-riding, the more leaders contribute, the more followers contribute. Our results were clearly not consistent with the equilibrium predictions, supporting the idea that leading by example positively influence group cooperation. The present study makes several contributions to the literature on leading by example.

First, this study conducted a cross-level validation, thus conceiving the possible integrative framework of both signaling and reciprocity perspectives on leading by example. Previously, two major theoretical streams developed competing theoretical explanations for leading by example, one by focusing on the group level and the other by focusing on the individual level. Those espousing the signaling perspective paid more attention to the information transferring, especially the presence of asymmetric information (Chen and Komorita, 1994; Hermalin, 1998; Dannenberg et al., 2014; Pereira and Lenaerts, 2017), which is a group level phenomenon. On the other hand, given the significant effect of sequentiality on reciprocal behavior, adherents of the reciprocity perspective prefer to focus on follower information processing (Chaudhuri and Paichayontvijit, 2006), which is on the individual level. Overall, the signaling perspective claims that committing effort first to signal information about the marginal return of cooperative behavior is the fundamental mechanism of leading by example, while the reciprocal perspective states that the cooperation of followers is purely determined by sequentiality and individual traits. In this study, a more integrated model encompassing different levels according to the empirical evidence suggests that the information structure and followers work together to make leading by example effective. Thus, the two existing theoretical perspectives provide an incomplete interpretation. It reminds us that a more comprehensive understanding of the mechanism of leading by example should be based on multilevel theoretical perspectives.

Second, three levels of leading by example have been compared in this study to reveal their effect on followers, working in a more systematic fashion in order to understand good and poor leaders. The existing literature mostly explored the positive effect of leading by example on follower cooperation (Hermalin, 1998; Gächter et al., 2012). To a large extent, scholars ignored the negative effect of poor leading by example, resulting in an incomplete diagram of follower responses toward leading by example. We find that high levels of leading by example have a positive impact on group cooperation, which functions by sacrificing leaders’ potential gains. This finding is in line with a previous study about what makes a good leader (Hermalin, 1998). Moreover, our findings that leaders with low levels of leading by example impede group cooperation, suggest that taking free rides from followers is a sign of a poor leader. Further analysis reminds us that a poor leading example negatively affects follower cooperation, which is even worse than in the control conditions. It opens a possibility to understand what makes a poor leader by discussing how low level leading by example exerts a negative influence on followers.

Third, this study supports the idea that individuals as information processers behave as “conditional cooperators” (Chaudhuri and Paichayontvijit, 2006). Several follower personal traits are proven to be associated with their cooperative behavior in the PGG with leading by example. An important implication is that the cooperation of followers is based on the sensitivity of followers toward how they perceive leading by example, suggesting that different individuals fit best into different leading by example paradigms. Thus, to sustain a high level of group cooperation, selecting appropriate group members is equally important to effective leading by example. To examine this idea, more empirical research is necessary.

Fourth, this study contributes to the research method by separating the complex interaction between leaders and followers during repeated trails. As information processers, leaders may change their contributions according to the cooperation followers. As a possible confounding variable, the interaction between leaders and followers is difficult to interpret and has barely been mentioned in previous research. To avoid confounding this relationship, this study simulates leader contribution from a prepared set. This manipulation helps create better experimental settings for exploring the mechanism of leading by example, which is instructive for further research.

### Limitations and Future Directions

There are several limitations to this study which need to be addressed in future research, as well as future directions. First, since leaders can choose either to take action or make a non-binding pledge, future research may try to employ leading by words in order to observe the mechanism of leadership (Dannenberg, 2015). Also, some experimental evidence to explain the varying effects of punishment and reward as different forms of leading by example is called for. Second, the PGG paradigm based on well-controlled experiments is limited in ecological validity. The reciprocity motivated by moral sentiments drives followers to react positively to the contributions of others, even if they believe that their decisions have no influence on the subsequent contributions of others (Figuieres et al., 2012). It can’t fully exclude the possibility of confusion between reciprocity to leaders and to other followers. There also could be several leaders instead of only one in our daily lives. It is reported that the increase of the stake size involved in the game causes individuals to be more likely to choose the rational selfish strategy (Zhang et al., 2018). Thus, the stake-size effect is also likely to moderate the effect of leading by example on group cooperation. Future research should be careful to apply conclusions drawn from experimentation in the real world. Many more empirical studies based on longitudinal design and field research methodology are needed to bring more robust evidence in support of the mechanisms revealed in this present study. Third, while data in this study is collected from China and cultural factors are barely associated with this topic, it is reasonable to speculate that culture plays an important role (Schein, 2015; Spector et al., 2015). Hence, cross-culture comparisons of leading by example would be helpful for exploring the mechanism of leadership. Fourth, this study merely addresses leading by example, which is a narrow aspect of leadership. Although these findings are instructive and suggest that effective leadership depends on leaders’ cooperativeness and followers’ individual differences, many more studies should be conducted to examine these conclusions in different contexts as well as in field studies.

In summary, this study used the PGG paradigm and HLM analysis to explore the effects of leading by example and individual traits on the cooperation of followers. This present study complements previous studies by separating leading by example from the reaction to the contributions of followers. It also provides compelling evidence for investigating the impact of leading by example and followers’ personal traits in a unified model. Furthermore, the results are in support of the conjecture that both signaling and reciprocity offer reasonable explanations but on different levels. Nevertheless, future research should take other cognitive and psychological factors into account to understand how groups benefit from leading by example.

## Author Contributions

HQ focused on the theoretical foundation, model development, research design, and data analysis. YZ focused on the research design, data collection, and data analysis. GH and ZW as the supervisor focused on the theoretical foundation.

## Conflict of Interest Statement

The authors declare that the research was conducted in the absence of any commercial or financial relationships that could be construed as a potential conflict of interest.
